# Antibacterial activity of natural spices on multiple drug resistant *Escherichia coli *isolated from drinking water, Bangladesh

**DOI:** 10.1186/1476-0711-10-10

**Published:** 2011-03-15

**Authors:** Shahedur Rahman, Anowar Khasru Parvez, Rezuanul Islam, Mahboob Hossain Khan

**Affiliations:** 1 Department of Biotechnology and Genetic Engineering, Islamic University, Kushtia-7003, Bangladesh; 2 Department of Microbiology, Jahangirnagar University, Savar, Dhaka-1342, Bangladesh

## Abstract

**Background:**

Spices traditionally have been used as coloring agents, flavoring agents, preservatives, food additives and medicine in Bangladesh. The present work aimed to find out the antimicrobial activity of natural spices on multi-drug resistant *Escherichia coli *isolates.

**Methods:**

Anti-bacterial potentials of six crude plant extracts (*Allium sativum*, *Zingiber officinale*, *Allium cepa*, *Coriandrum sativum*, *Piper nigrum *and *Citrus aurantifolia*) were tested against five *Escherichia coli *isolated from potable water sources at kushtia, Bangladesh.

**Results:**

All the bacterial isolates were susceptible to undiluted lime-juice. None of them were found to be susceptible against the aqueous extracts of garlic, onion, coriander, pepper and ginger alone. However, all the isolates were susceptible when subjected to 1:1:1 aqueous extract of lime, garlic and ginger. The highest inhibition zone was observed with lime (11 mm).

**Conclusion:**

Natural spices might have anti-bacterial activity against enteric pathogens and could be used for prevention of diarrheal diseases. Further evaluation is necessary.

## Introduction

Multi-drug resistant strains of *Escherichia coli *are widely distributed in hospitals and are increasingly being isolated from community [1]. Thus, it is urgent need to find out new antimicrobial agents. However, new families of antimicrobial agents will have a short life expectancy [2]. For this reason, researchers are increasingly turning their attention to herbal products, looking for new leads to develop better drugs against multidrug resistant microbe strains [3].

Spice plants and essential oils extracted from them have become important due to their potential antimicrobial [4-6] and stimulating effects in the animal digestive system. The antimicrobial effectiveness of mustard, clove, cinnamon and their essential oils were reported for the first time around 1880's. Antimicrobial effectiveness of spices depend on the kind of spice, its composition and concentration, type and concentrations of the target microorganism, substrate composition, and processing and food storage conditions [7]. Spice plants have been used traditionally as coloring agents, flavoring agents, preservatives, food additives and as well as antiparasitic, antihelmintic, analgesic, expectorant, sedative, antiseptic and anti-diabetic substances in many parts of the world [8]. In addition, they possess biological activities such as that of antioxidants [9] and hypocholesterolemics [10]. In this study, we aimed to explore the antibacterial effect of natural spices on multiple drug resistant *E. coli *isolates.

## Materials and methods

Multi-drug resistant *E. coli *used in present study were isolated from drinking water of various region of kushtia town by standard methods [11]. Pure cultures of isolates were preserved at 4°C on nutrient agar slants. In order to confirm the identity of the test bacterial isolates morphological characteristics and conventional biochemical tests were performed according to Harley and Prescott [11]. The spices were bought from local market. Garlic and onion were peeled, cut into pieces and sun-dried for one week. These were then grounded using a blender. Twenty gram of the ground material (garlic, onion, ginger, pepper and coriander seed) were soaked in 100 ml of hot sterile water and allowed to stand for 72 h. The lime fruits were washed with sterile water then cut open with a sterile knife and the juice pressed out. The crude extracts were obtained by filtration [12]. Sterile paper disks of 5 mm diameter were impregnated with 10 μl of crude extracts and mixtures of different crude extracts (1:1, 1:1:1), then dried in a hot air oven at 60°C for five min. Each disk contains approximately 2 μg of crude extract. All extracts were stored at 4°C when not in use.

## Susceptibility testing

Standard norfloxacin (NOR, 10 μg/disc), clindamycin (CC, 2 μg/disc), neomycin (N, 30 μg/disc), cefoxitin (FOX, 30 μg/disc), streptomycin (S, 10 μg/disc), ciprofloxacin (CPX, 5 μg/disc), chloramphenicol (C, 30 mcg/disc), tetracycline (T, 30 mcg/disc), penicillin G (P, 10 units/disc), erythromycin (E, 15 mcg/disc), cefotaxime (CTX, 30 μg/disc), nalidixic acid (NA, 30 mcg/disc), cloxacillin (CX, 1 mcg/disc), kanamycin (K, 30 mcg/disc), amoxicillin (AM, 30 mcg/disc) were used for comparison of the antibacterial activity. *In vitro *antibacterial susceptibility of the test samples was carried out by disc diffusion method [13,14]. The antibacterial activities were determined by measuring the diameter of the zone in mm.

## Results and discussion

Table 1 shows the antibiotic resistance pattern of the isolates. All strains showed resistance against the activity of clindamycin and cloxacillin. Penicillin G is also not quite effective against those isolates as four of the isolates are resistant against it. Highest growth inhibition zones were found with ciprofloxacin followed by cefotaxime and norfloxacin. The multiple antibiotic resistance of *E. coli *demonstrated in this study accord with those found in other studies [15-17].

**Table 1:** Antibiotic resistant pattern of the isolated strains

	Growth inhibition zone in millimeter														
	**NOR**	**CC**	**N**	**FOX**	**S**	**CPX**	**C**	**T**	**P**	**E**	**CTX**	**NA**	**CX**	**K**	**AM**

**Isolate 1**	22	-	14	18	16	20	19	19	-	14	25	17	-	17	18
**Isolate 2**	27	-	15	21	20	35	26	24	-	10	31	26	-	20	26
**Isolate 3**	26	-	15	21	17	30	23	25	7	14	27	20	-	17	26
**Isolate 4**	30	-	16	22	10	34	30	17	-	18	30	22	-	20	-
**Isolate 5**	29	-	15	21	21	30	27	25	-	20	34	25	-	20	25

Note: "-" = Negative test, NOR = Norfloxacin (10 μg/disc), CC = Clindamycin (2 μg/disc), N = Neomycin (30 μg/disc), FOX = Cefoxitin (30 μg/disc), S = Streptomycin (10 μg/disc), CPX = Ciprofloxacin (5 μg/disc), C = Chloramphenicol (30 mcg/disc), T = Tetracycline (30 mcg/disc), P = Penicillin G (10 units/disc), E = Erythromycin (15 mcg/disc), CTX = Cefotaxime (30 μg/disc), NA = Nalidixic acid (30 mcg/disc), CX = Cloxacillin (1 mcg/disc), K = Kanamycin (30 mcg/disc), AM = Amoxicillin (30 mcg/disc).

Table 2 shows that the aqueous extracts of *Allium sativum *and *Zingiber officinale *alone and in combination (1:1) did not exhibit any *in vitro *inhibition on the growth of test organisms except in case of one isolate for later cases (7 mm). Similarly *Allium cepa*, *Piper nigrum*, and *Coriandrum sativum *seed alone did not exhibit any *in vitro *anti-bacterial effect, however the combination of *Allium cepa *+ *Allium sativum *(1:1) and *Citrus aurantifolia *+ *Zingiber officinale *+ *Allium sativum *(1:1:1) showed inhibition zones. *Citrus aurantifolia *juice showed the highest effect on the test organisms. These findings are in good accord with others [12,18-22].

**Table 2:** Diameter (mm) of zone of inhibition produced by various spice extracts

Isolated bacterial strains	1	2	3	4	5	6	7	8	9
**Isolate 1**	-	-	-	-	-	-	-	8	10
**Isolate 2**	-	-	-	-	-	**7**	8	9	10
**Isolate 3**	-	-	-	-	-	-	7	9	9
**Isolate 4**	-	-	-	-	-	-	-	7	8
**Isolate 5**	-	-	-	-	-	-	-	10	11

Note: "-" = No inhibition zone, 1 = *Zingiber officinale *(Aqueous extracts), 2 = *Allium cepa *(Aqueous extracts), 3 = *Allium sativum *(Aqueous extracts), 4 = *Piper nigrum*, (Aqueous extracts), 5 = *Coriandrum sativum*, seed (Aqueous extracts), 6 = *Zingiber officinale *+ *Allium sativum *(1:1 aqueous extracts), 7 = *Allium cepa *+ *Allium sativum *(1:1 aqueous extracts), 8 = *Citrus aurantifolia *+*Zingiber officinale *+ *Allium sativum *(1:1:1 aqueous extracts), 9 = *Citrus aurantifolia*.

*A. sativum *has traditional dietary and medicinal applications as an anti-infective agent [23]. *In vitro *evidence of the antimicrobial activity of fresh and freeze-dried garlic extracts against many bacteria [24], fungi and viruses [25] supports these applications. Allicin, the active ingredient of *A. sativum*, acts by partially inhibiting DNA and protein synthesis and also totally inhibiting RNA synthesis as a primary target [26]. Like *A. sativum*, *A. cepa *is another medicinally important anti-infective agent [12]. Raw *A. cepa *can completely sterilize mouth and throat. Organosulfur compounds (OSCs) and phenolic compounds have been reported to be involved in the *A. cepa *antimicrobial activity [27]. *Z. officinale *has been used widely as herbal medicine. In particular, its gingerol-related components have been reported to possess antimicrobial and anti fungal properties, as well as several pharmaceutical properties [28]. *C. sativum *is considered both herb and spice since both its leaves and seeds are used as a seasoning condiment [18]. It has traditionally been referred to as antimicrobial [29]. The seeds of *C. sativum *contain 0.5-1% essential oil and are rich in beneficial phytonutrients including carvone, geraniol, limonene, borneol, camphor, elemol and linalool [30]. Ferrous sequestering activity of these compounds may play role in inhibiting microbial growth [31]. *P. nigrum *is used to treat asthma, chronic indigestion, colon toxins, obesity, sinus, congestion, fever, intermittent fever, cold extremities, colic, gastric ailments and diarrhoea [32]. It has shown to have antimicrobial activity [33]. From HPTLC analysis it is reported that the major phytochemical present in the crude extract of *P. nigrum *was found to be piperine thus the inhibitor effect of crude extract of *P. nigrum *and its active constituent was found to be piperine [34]. *C. aurantifolia *is popular as fruit and food ingredient for flavoring and adding acidity, its juices have been reported to exhibit antibacterial activity against wide range of microbes including *Klebsiella pneumoniae, Shigella flexnerii, E. coli *ATCC 25922 and *Vibrio cholerae *[35,36].

The antimicrobial potency of plants is believed to be due to tannins, saponins, phenolic compounds, essential oils and flavonoids [37]. It is interesting to note that even crude extracts of these plants showed good activity against multidrug resistant strains where modern antibiotic therapy has limited effect.

The effect of these spices on these organisms *in vivo *cannot be predicted from this study. And though paper disk assays a practical approach to study potential antibacterial compounds, using the size of inhibition zone to indicate relative antibacterial activity is not adequate. The zone must be affected by the solubility and rate of diffusion in agar medium or its volatilization; and thus the results could be affected.

Thus, there is a need for detailed scientific study of traditional medical practices to ensure that valuable therapeutic knowledge of some plants is preserved and also to provide scientific evidence for their efficacies.

## Conclusions

The extracts of these spices could be a possible source to obtain new and effective herbal medicines to treat diarrheal diseases caused by multi-drug resistant strains of *E. coli *in community. However, it is necessary to isolate the active constituents, and determine their toxicity, side effects and pharmaco-kinetic properties.

## Authors' contributions

MSR conducted laboratory analysis and drafted the manuscript. MSR and MAKP contributed equally to this work. MAKP, MRI and MMHK participated in the design of the study and supervise the work. All authors were involved in the interpretation of the data and approved the final manuscript.

## Competing interests

The authors declare that they have no competing interests.
